# Testosterone hormone levels and breast muscle performance of Pelung chickens after zinc sulfate and synthetic testosterone supplementation

**DOI:** 10.14202/vetworld.2024.2365-2369

**Published:** 2024-10-27

**Authors:** Rizki Fitrawan Yuneldi, Claude Mona Airin, Hendry T. S. Saragih, Andhika Yudha Prawira, Pudji Astuti

**Affiliations:** 1 Research Center for Applied Zoology, Research Organization for Life Science and Environment, National Research and Innovation Agency (BRIN), Cibinong, Indonesia; 2 Department of Physiology, Faculty of Veterinary Medicine, Universitas Gadjah Mada, Yogyakarta, Indonesia; 3 Laboratory of Animal Development Structure, Faculty of Biology, Universitas Gadjah Mada, Yogyakarta, Indonesia

**Keywords:** muscle, Pelung chicken, performance, testosterone, texture

## Abstract

**Background and Aim:**

Pelung chicken *Gallus gallus gallus* (Linnaeus, 1758) is a chicken endemic to Cianjur, West Java, Indonesia. This study aimed to determine the effects of zinc sulfate (ZnSO_4_) and synthetic testosterone supplementations for 56 days on testosterone hormone levels and breast muscle performance in Pelung chickens.

**Materials and Methods:**

This study used 12 Pelung chickens with three treatment groups (G) and four replications, namely, control (G0), ZnSO_4_ 0.9 mg/kg (G1), and synthetic testosterone 3 mg/day (G2). Chickens were acclimatized for 7 days and then supplemented for 56 days. Drinking water and commercial standard feed were provided ad libitum. Blood was collected through the brachial vein for the analysis of testosterone levels using the enzyme-linked immunosorbent assay. The samples were collected every 14 days; on days 0, 14, 28, 42, and 56. Breast muscles were collected for texture analysis, and breast muscle preparations were made with hematoxylin-eosin staining to measure the fascicle area (FA), number of myofibers in one fascicle (NMOF), and myofiber area (MA). The collected data were analyzed using analysis of variance at a 95% confidence level with the help of Statistical Package for the Social Sciences v.29.0. Furthermore, principal component analysis (PCA) was performed with the help of Minitab v. 19.

**Results:**

Statistical analysis results on 56 days of testosterone level parameters showed that G2 was significantly different from all treatments (p < 0.05). The results of statistical analysis on Pelung chicken breast muscle performance, especially hardness, chewiness, FA, NMOF, and MA, were significantly different (p < 0.05) compared with the other treatments. The results of PCA showed that the testosterone level parameters were positively correlated with FA, NMOF, MA, hardness, and chewiness, whereas the fracture parameters were negatively correlated with all parameters except the springiness index and were significantly different between the G2 group and the other groups (p < 0.05).

**Conclusion:**

It can be concluded that supplementing synthetic testosterone 3 mg/day body weight for 56 days can improve testosterone levels and breast muscle performance, especially hardness, chewiness, FA, NMOF, and MA in Pelung chickens.

## Introduction

Pelung chicken *Gallus gallus gallus* (Linnaeus, 1758) is a native Indonesian chicken originating from Cianjur, West Java, Indonesia. This chicken has the distinction of a long and distinctive voice [1, 2]. Furthermore, its body is tall and large, and it has a relatively high body weight (BW) [3]. Breast muscle performance is influenced by the hormone testosterone [4]. It controls voice, growth, muscle strength, spermatogenesis, and sex drive [3, 5–11]. Parameters for determining muscle performance include muscle strength, apart from measuring chest circumference, breast muscle weight, fascicle area (FA), myofiber area (MA), number of myofiber in one fascicle (NMOF), and the percentage of positive proliferating cell nuclear antigen can also be supported by texture parameters in Pelung chicken breast muscles by measuring hardness, tenderness, springiness index, chewiness, and fracture [4, 12]. Injection of anabolic hormones, such as testosterone, into male poultry, is often performed to improve their performance [13].

The testosterone in the body is naturally converted into estrogen or estradiol by aromatase enzyme, which causes challenges in maintaining stable high testosterone levels [14]. However, continuous injection of low-dose synthetic testosterone can cause downregulation mechanisms and drastically reduce testosterone levels [13]. During downregulation, testosterone receptors decrease, leading to decreased voice performance [13, 15]. In addition, it can cause atrophy of the seminiferous tubules in Pelung chickens [4]. According to Yuneldi *et al*. [9], administration of testosterone 0.1 mL a day for 35 days can increase testosterone levels but reduce testicular weight significantly compared with controls and those administered blood clamshell powder (*Anadara granosa*) as a natural aromatase blocker at a dose of 0.036 mg/40 g BW. However, zinc sulfate and synthetic testosterone supplementation on breast muscle performance, especially texture of breast muscle in Pelung chickens has not been investigated.

Therefore, this study aimed to evaluate the effects of administering zinc sulfate (ZnSO_4_) and synthetic testosterone supplementation for 56 days on testosterone levels and breast muscle performance in Pelung chickens.

## Materials and Methods

### Ethical approval

All research procedures were approved by the Ethics Committee of Integrated Testing and Research, Universitas Gadjah Mada (UGM) (Approval no. 00020/04/LPPT/V/2020).

### Study period and location

The study was conducted from April to September 2022 at a Pelung chicken farm in Bantul, Yogyakarta. Texture profile analysis and hematoxylin-eosin (HE) staining of Pelung chicken breast muscle were conducted in the Faculty of Animal Science, Faculty of Agricultural Technology and Animal Development Structure Laboratory, Faculty of Biology, UGM. Testosterone level analysis was conducted at the Physiology Laboratory, Faculty of Veterinary Medicine, UGM, Indonesia.

### Experimental design

Before treatment, Pelung chickens were allowed to acclimatize for 7 days, and their health was monitored daily. The chicken housings were individual-stage wood cages with slit bases (60 × 60 × 60 cm). Drinking water and commercial standard feed were provided *ad libitum* [16]. The study used 12 male Pelung chickens (the sample size was small as the chicken falls under the threatened species category) aged 40–56 weeks. The sample size was determined as described by Yuneldi *et al*. [4]. The general physiological performance of male Pelung chickens decreased from 40 to 56 weeks. Pelung chickens (BW ± 3 kg) were divided randomly into three treatment groups (G): G0 (control); G1 (ZnSO_4_ at 0.9 mg/kg BW); and G2 (synthetic testosterone 3 mg/day). Each treatment group contained four Pelung chickens. ZnSO_4_ was administered orally [3, 6, 9, 17] and testosterone was administered subcutaneously [9, 18]. The chickens were supplemented for 56 days.

### Testosterone analysis

Testosterone hormone level analysis was carried out according to the procedure described by Yuneldi *et al*. [3]. Serum samples were examined for testosterone hormone levels using enzyme-linked immunosorbent assay (ELISA). Testosterone level analysis was performed according to the manufacturer’s instructions in the kit (Calbiotech^®^, California, USA) using competitive ELISA. Absorbance was analyzed using ELISA or a microplate reader (Zenix-320, Shenzhen, China) at an absorbance of 450 nm for 15 min.

### Euthanasia and organ preparation

On the 56^th^ day, chickens were slaughtered using the halal method. Breast muscles were collected and washed with 0.9% NaCl [19, 20]. The left breast muscle was analyzed for tenderness, and the right muscle was analyzed for hardness, springiness index, chewiness, and fracture. Furthermore, part of the right breast muscle was cut to a smaller size of approximately 1 × 1 cm and fixed for 18–24 h using 10% neutral buffer formalin solution before histological preparation [21, 22].

### HE staining method

The process of making slides using the paraffin method, after cutting them to 5 μm, was performed and placed on a glass object. Furthermore, HE staining was performed (Merck, Darmstadt, Germany), and the slides were observed under a microscope (Leica DM 750, Germany) at a magnification of 10 × 10. The area of myofiber and fascicle was measured, and the NMOF was calculated. These measurements were performed using ImageJ software (Version 1.53 k, National Institute of Health, USA) [23].

### Texture analysis of breast muscle

Pelung chicken breast muscle analysis with the parameter of tenderness (kg/cm^2^) was performed using a Warner-Bratzler (WB) device, and the parameters of hardness (N), springiness index, chewiness (N), and fracture (N) were tested using a Texture Analyzer TA1 (Ametek Lloyd Instruments Ltd., Fareham, UK). Tenderness analysis was continued by testing meat measuring 1.5 × 0.67 cm in length parallel to the muscle fibers. Changes in the scale of the WB tool indicate tenderness scores. The analysis of hardness (N), springiness index, chewiness (N), and fracture (N) requires a meat block with a minimum size of 1 × 1 × 1 cm. Operation of the Texture Analyzer and data obtained using Nexygenplus software (Ametek Lloyd Instrument Ltd.).

### Statistical analysis

All parameters were statistically analyzed by one-way analysis of variance (ANOVA) using Statistical Package for Social Sciences v.29.0 (IBM, NY, USA) with a 95% confidence level (α = 0.05). The analysis was confirmed using Duncan’s test [4, 24]. Furthermore, principal component analysis (PCA) was applied. All the data were analyzed statistically using PCA in Minitab® statistical software v.19 (Minitab Inc., State College, PA, USA) to reduce dimensionality and identify correlations between all parameters. Two principal components (PC) with cumulative proportion values exceeding 70% were plotted in the graphic as loading plots of variables and score plots of objects.

## Results

### Testosterone hormone levels and breast muscle performance in Pelung chickens

**Figure-1:**
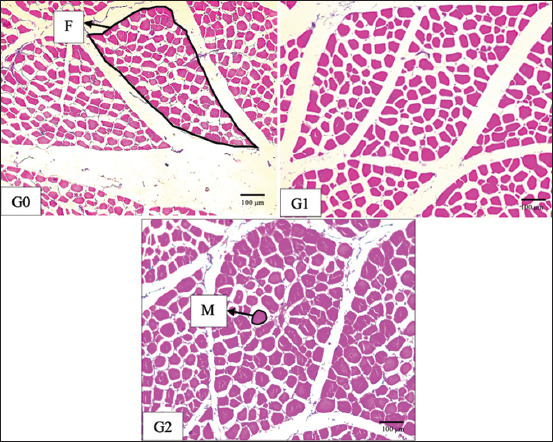
Histology of the breast muscle of male Pelung chickens aged 40–56 weeks after supplementation for 56 days. G0=Control, G1=ZnSO_4_ 0.9 mg/kg body weight, G2=Synthetic testosterone 3 mg/day. Magnification 10 *×* 10. Hematoxylin-eosin staining. F=Fascicle, M=Myofiber, G=Group.

The results of ANOVA for testosterone hormone levels showed that there was a significant difference (p < 0.05) in G2 compared with other treatments on days 28, 42, and 56 (Table-1). The results of the ANOVA statistical analysis of hardness and chewiness showed that the value for G2 was higher (p < 0.05) compared with the other treatments. However, the springiness index, fracture, and tenderness showed no significant differences between the treatments (p > 0.05) (Table-2). The FA, NMOF, and MA were significantly different (p < 0.05) in G2 compared with the other treatments (Table-3 and Figure-1).

**Table-1:** Testosterone levels of male Pelung chickens aged 40–56 weeks after 56 days of supplementation (Average ± standard deviation).

Group	0 day (ng/mL)	14 day (ng/mL)	28 day (ng/mL)	42 day (ng/mL)	56 day (ng/mL)
G0	0.19 ± 0.09^a^	0.25 ± 0.12^b^	0.30 ± 0.05^c^	0.38 ± 0.04^b^	0.80 ± 0.07^b^
G1	0.44 ± 0.38^a^	0.50 ± 0.28^ab^	0.52 ± 0.15^b^	0.65 ± 0.06^b^	0.97 ± 0.10^b^
G2	0.74 ± 0.45^a^	0.89 ± 0.49^a^	1.47 ± 0.19^a^	4.70 ± 0.41^a^	9.94 ± 0.18^a^
p-value	0.052	0.042	<0.001	<0.001	<0.001

^a–c^Means with different superscripts within the same column are significantly different (p < 0.05). G0=Control, G1=ZnSO_4_ 0.9 mg/kg body weight, G2=Synthetic testosterone 3 mg/day. SD=Standard deviation

**Table-2:** Breast muscle texture of male Pelung chickens aged 40–56 weeks after 56 days of supplementation (Average ± standard deviation).

Group	Tenderness (kg/cm^2^)	Hardness (N)	Springiness index	Chewiness (N)	Fracture (N)
G0	4.06 ± 0.93^a^	134.43 ± 12.10^b^	0.667 ± 0.01^a^	29.08 ± 13.39^b^	2.08 ± 0.09^a^
G1	3.82 ± 1.49^a^	139.77 ± 28.72^b^	0.685 ± 0.03^a^	31.13 ± 6.26^b^	2.01 ± 0.13^a^
G2	4.17 ± 0.10^a^	190.70 ± 32.42^a^	0.687 ± 0.03^a^	93.50 ± 20.98^a^	2.04 ± 0.14^a^
p-value	0.884	0.025	0.612	<0.001	0.731

^a–c^Means with different superscripts within the same column are significantly different (p < 0.05). G0=Control, G1=ZnSO_4_ 0.9 mg/kg body weight, G2=Synthetic testosterone 3 mg/day. SD=Standard deviation

**Table-3:** Myofibers in one fascicle of Pelung chickens aged 40–56 weeks after 56 days of supplementation (Average ± standard deviation).

Group	Fascicle area (µm^2^)	NMOF	Myofiber area (µm^2^)
G0	210551.34 ± 13147.80^c^	100.75 ± 2.09^c^	1937.74 ± 56.90^c^
G1	279314.87 ± 14349.24^b^	107.58 ± 2.10^b^	2123.52 ± 83.75^b^
G2	390620.03 ± 7460.67^a^	126.51 ± 3.42^a^	3604.32 ± 286.58^a^
p-value	<0.001	<0.001	<0.001

^a–c^Means with different superscripts within the same column are significantly different (p < 0.05). G0=Control, G1=ZnSO_4_ 0.9 mg/kg body weight, G2=Synthetic testosterone 3 mg/day. SD=Standard deviation, NMOF=Number of myofibers in one fascicle

According to PC analysis, several parameters were clustered into one main cluster (Cluster I) and two other clusters (Cluster II and III). The main cluster (Cluster I) consisted of testosterone level, FA, MA, NMOF, hardness, and chewiness, with a highly positive correlation. Cluster II consisted of fracture and springiness, whereas Cluster III consisted of tenderness. Fracture was negatively correlated with Clusters I and III, which means that increasing the value in Clusters I and III will decrease the value of fracture. The score plot of the object revealed that G0, G1, and G2 were separated from each other, with G2 being plotted as having the highest score. Therefore, G2 generally obtained the highest value for almost all parameters compared to G0 and G1.

## Discussion

### Testosterone hormone levels in Pelung chickens at 56 days post-supplementation

This study showed that supplementing synthetic testosterone 3 mg/day increased testosterone hormone levels in Pelung chickens. This is because testosterone, which is injected continuously every day, accumulates in the blood, and testosterone levels in the blood continue to increase until the 56^th^ day. This is in accordance with the findings of Yuneldi *et al*. [9], who reported that supplementing testosterone 0.1 mL/day for 35 days can increase testosterone levels in male-layer chickens. This is due to the direct intake of testosterone into the blood, which causes testosterone levels to increase.

However, continuous testosterone injections cause downregulation, such as a drastic reduction in testicular weight, compared with other treatments [9]. Therefore, injection of anabolic hormones such as testosterone into male poultry is not recommended for maintaining performance. Huang *et al*. [25] showed that continuous administration of testosterone can cause downregulation. Direct testosterone injection can induce the downregulation of these mechanisms [15, 26]. Yuneldi *et al*. [4] reported that the administration of testosterone at 3 mg/day for 56 days caused atrophy of the seminiferous tubules in Pelung chickens.

### Performance of breast muscle in Pelung chickens at 56-day post-supplementation

The results showed a significant increase in hardness and chewiness in the breast muscles of Pelung chickens, but an increase did not follow this trend in springiness index, fracture, and tenderness. In contrast to the results of texture analysis of Bangkok chicken breast muscle, a combination of clam shell powder and milkfish bone powder increased the springiness index compared with hardness and tenderness [12]. The hardness and chewiness results increased in line with the fascicle, NMOF, and MAs, resulting in the G2 treatment, which was significantly different from the other treatment groups. These results are similar to those reported by Yuneldi *et al*. [4] in that the NMOF, MA, and fasciculus area experienced significant differences in treatment given synthetic testosterone. These results are interconnected in that the myofiber and FAs increase with the increasing hardness of NMOF. According to U-Chupaj *et al*. [27], muscle hardness is related to the number of myofiber in the muscle. High hardness is related to the density, number, and composition of muscle myofiber in chickens [28]. The neuromuscular system activates more myofiber, leading to stronger muscle contraction produced [29]. High hardness indicates a strong muscle structure and the number of myofiber [12].

## Conclusion

Supplementing synthetic testosterone 3 mg/day BW for 56 days can increase testosterone hormone levels and breast muscle performance, especially in the hardness, chewiness, FA, NMOF, and MA of Pelung chickens. Therefore, future study is needed regarding synthetic testosterone residues in breast muscle.

## Authors’ Contributions

PA, CMA, HTSS, and RFY: Planned and designed the study. PA, CMA, HTSS, and AYP: Supervised the study. PA, CMA, and RFY: Collected and analyzed the samples. PA, CMA, HTSS, RFY, and AYP: Drafted and revised the manuscript. All authors have read and approved the final manuscript.
